# High-sensitivity cardiac troponin I and risk of heart failure in patients with suspected acute coronary syndrome: a cohort study

**DOI:** 10.1093/ehjqcco/qcx022

**Published:** 2017-07-19

**Authors:** Dominik Stelzle, Anoop S V Shah, Atul Anand, Fiona E Strachan, Andrew R Chapman, Martin A Denvir, Nicholas L Mills, David A McAllister

**Affiliations:** 1 BHF Centre for Cardiovascular Science, University of Edinburgh, 49 Little Fracne Cresc, Edinburgh EH16 4SB, UK; 2 The Institute of Health and Wellbeing, University of Glasgow, Glasgow, UK; 3 The Usher Institute of Population Health and Informatics, University of Edinburgh, Edinburgh, UK

**Keywords:** High-sensitivity cardiac troponin, Acute coronary syndrome, Heart failure

## Abstract

**Aims:**

Heart failure may occur following acute myocardial infarction, but with the use of high-sensitivity cardiac troponin assays we increasingly diagnose patients with minor myocardial injury. Whether troponin concentrations remain a useful predictor of heart failure in patients with acute coronary syndrome is uncertain.

**Methods and results:**

We identified all consecutive patients (*n* = 4748) with suspected acute coronary syndrome (61 ± 16 years, 57% male) presenting to three secondary and tertiary care hospitals. Cox-regression models were used to evaluate the association between high-sensitivity cardiac troponin I concentration and subsequent heart failure hospitalization. C-statistics were estimated to evaluate the predictive value of troponin for heart failure hospitalization. Over 2071 years of follow-up there were 83 heart failure hospitalizations. Patients with troponin concentrations above the upper reference limit (URL) were more likely to be hospitalized with heart failure than patients below the URL (118/1000 vs. 17/1000 person years, adjusted hazard ratio: 7.0). Among patients with troponin concentrations <URL the rate of heart failure hospitalization was 2.80-fold higher [95% confidence interval (95% CI 1.81–4.31)] per doubling of troponin concentration. On adding troponin to a model with demographic, cardiovascular risk factor, and clinical variables, the prediction of heart failure hospitalization improved considerably (C-statistic 0.80 vs. 0.86, *P* < 0.001).

**Conclusion:**

Cardiac troponin is an excellent predictor of heart failure hospitalization in patients with suspected acute coronary syndrome. The strongest associations were observed in patients with troponin concentrations in the normal reference range, in whom high-sensitivity cardiac troponin assays identify those at increased risk of heart failure who may benefit from further investigation and treatment.

## Introduction

Heart failure is common, important, and costly. More than 15 million people are thought to have heart failure in Europe.^1^ It is estimated that in the USA alone the total direct costs of heart failure care is more than $20 billion *per annum*.^2^ Despite improvements in diagnosis and the development of effective therapies for patients with heart failure, the case-fatality rate at 5-years is 50%.^3–6^ One of the major causes of heart failure is acute myocardial infarction with symptoms developing in those patients who have sustained significant myocardial injury and ventricular impairment.^7^^,^^8^ However, with the development of high-sensitivity cardiac troponin assays we increasingly identify patients with minor myocardial injury.^9–12^ Whether cardiac troponin concentration remains a useful predictor in this group of patients is uncertain. Furthermore, high sensitivity cardiac troponin assays are increasingly used to risk-stratify patients to identify those likely to benefit from hospitalization and/or further investigation.^13^ We therefore examined the association and predictive utility of cardiac troponin concentration for subsequent admission to hospital with heart failure in consecutive patients with suspected acute coronary syndrome.

## Methods

We included 4748 consecutive patients who presented to the Emergency Department with suspected acute coronary syndrome to three secondary and tertiary hospitals in Edinburgh, Scotland between 1 June 2013 and 31 January 2014 and who survived to discharge. These patients were enrolled in the standard care arm of a stepped-wedge cluster randomized controlled trial (ClinicalTrials.gov registration NCT01852123). All patients who had cardiac troponin requested by the attending clinician for suspected acute coronary syndrome were included. Patients were excluded if they had been admitted previously during the study period or did not reside in Scotland.^13^ The study was performed with the approval of the National Research Ethics Committee, and in accordance with the Declaration of Helsinki.

### High-sensitivity cardiac troponin I assay

The ARCHITECT_STAT_ high-sensitive troponin I assay (Abbott Laboratories, Abbott Park, IL, USA) was used to measure cardiac troponin I concentration in all patients. The limit of detection (LoD) of this assay is 1.2 ng/L and the upper reference limit (URL) or 99% centile in women is 16 and 34 ng/L in women and men, respectively.^13^ The lowest cardiac troponin I concentration with an inter-assay coefficient of variation (CV) of less than 10% was 4.7 ng/L according to the manufacturer and was 6 ng/L in our laboratory.^14^^,^^15^ In patients with more than one measure of cardiac troponin, the highest concentration was used. Values below the LoD were assigned a value of 1.2 ng/L (*n* = 506, 10.7%).

### Classification of myocardial infarction

The electronic patient record system TrakCare (InterSystems Corporation, Cambridge, MA, USA) was used to acquire baseline characteristics for each patient, including cardiovascular risk factors and past medical events.^13^ Hyperlipidaemia and hypertension were defined as a documented history of the condition, or by the respective use of lipid-lowering or anti-hypertensive medication. Smoking was defined as current or ex-smoking at admission. Killip class was determined by the attending clinician. Myocardial infarction type was classified by two investigators (A.S., A.A.) independently using the Third Universal Definition of Myocardial Infarction,^16^ with discrepancies being adjudicated by third investigator (N.M.). Patients were classified using all available clinical information at the time of the index admission (including peak troponin and serial change in troponin when these were available). Type 1 myocardial infarction was identified when myocardial necrosis occurred in the context of a presentation with suspected acute coronary syndrome with chest pain or evidence of myocardial ischaemia on the electrocardiogram. Patients with symptoms and signs of myocardial ischaemia on the electrocardiogram that were thought to be due to increased oxygen demand or decreased supply (e.g. tachyarrhythmia, hypotension, or anaemia) and myocardial necrosis were classified as having a type 2 myocardial infarction. Myocardial injury was defined as evidence of myocardial necrosis in the absence of any clinical features of myocardial ischaemia. A detailed description of the classification criteria was published elsewhere.^13^

### Outcomes

Outcomes were censored on 31 March 2014 (median follow-up 156 days, interquartile range 98–218 days). Hospitalization and death during the follow-up period were obtained via linkage to the national hospital database (‘the Scottish Morbidity Record’, SMR01). Heart failure was defined as any hospitalization assigned the code I50. All heart failure diagnoses were adjudicated by a physician and cardiologist independently (D.S., A.S.). Criteria for the adjudication of heart failure were based on review of inpatient clinical records and based on symptoms of congestive cardiac failure supported by imaging evidence of left ventricular dysfunction.

Agreement was 100% between the two investigators. Cardiac death was defined as deaths due to myocardial infarction, arrhythmia, or heart failure (ICD-10 codes I21/22 and I46-50).

### Statistical analysis

For all analyses, patients who died during the index admission were excluded. Analyses were performed for cardiac troponin I concentration as categorical and continuous variable. Patients were categorized into five groups according to the highest measured cardiac troponin I concentration. Group 5 included all patients who had cardiac troponin concentrations above the sex-specific 99th centile URL (>34 ng/L for men and >16 ng/L for women).^13^^,^^17^ The remaining patients were split into quartiles (Groups 1–4) by sex: 1.2–2.0, 2.1–4.0, 4.1–8.0, and 8.1–34.0 ng/L for men; 1.2–1.9, 2.0–2.9, 3.0–6.0, and 6.1–16.0 ng/L for women. These categories were assigned prior to analysing associations between groups and any of the study outcomes. For the analysis as a continuous variable, cardiac troponin concentrations were log-transformed as a linearizing transformation. Summary statistics were obtained for baseline characteristics by category. Hazard ratios (HRs) for first subsequent *hospitalization with heart failure* and for first subsequent *hospitalization with heart failure or cardiac death* were estimated according to group in Cox regression models adjusting for age, sex, cardiovascular risk factors (diabetes mellitus, hypertension, ischaemic heart disease, and previous myocardial infarction), and clinical features (systolic blood pressure and creatinine concentration). As there were neither heart failure hospitalizations nor cardiac deaths in the two groups with the lowest cardiac troponin concentrations, these two groups were collapsed. Similar models were used to estimate associations for troponin (log-transformed) as a continuous variable. Polynomial terms and penalized splines were used to present the associations, which were non-linear, in tables and figures, respectively.

Sensitivity analyses were performed having excluded patients with heart failure at the index presentation. Subgroup analyses of log-troponin concentration were conducted for patients with cardiac troponin concentrations below and above the 99th centile URL. Patients with elevated cardiac troponin concentrations were additionally analysed according to the diagnosis at the index presentation: type 1 myocardial infarction, type 2 myocardial infarction, and myocardial injury. The discrimination of troponin (with and without additional variables) was estimated using area under the receiver operating characteristic curves (C-statistics). Confidence intervals (CIs) for the C-statistics were obtained via bootstrapping.^18^ Analyses were performed in IBM SPSS Statistics Version 22.0.0 (Armonk, NY: IBM, USA, 2014) and R Version 3.0.1 (R project for statistical computing, Vienna, Austria).

## Results

There were 4870 patients with suspected acute coronary syndrome (mean age 61 ± 16 years, 57% men) enrolled between 1 June 2013 and 31 January 2014. One hundred and twenty-two patients died during the index presentation and these patients were excluded from this analysis. The median cardiac troponin concentration was 5 ng/L (interquartile range 2–22 ng/L). There were 1151 (24%) patients with cardiac troponin concentrations above the URL (Group 5); 723 (15.2%) patients were classified as having type 1 myocardial infarction, 158 (3.3%) type 2 myocardial infarction, and 270 (5.7%) with myocardial injury.

Patients with higher cardiac troponin concentrations were older, had a higher Killip class, and more cardiovascular risk factors. They were also more likely to have been treated with angiotensin converting enzyme inhibitors, angiotensin receptor blockers or beta blockers, and had higher creatinine concentrations (*Table 1*).

**Table 1:** Baseline characteristics of patients with suspected acute coronary syndrome stratified by cardiac troponin concentration

	Patients stratified by peak troponin concentration				
	Group 1	Group 2	Group 3	Group 4	Group 5^a^
	*n* = 900 (19%)	*n* = 899 (19%)	*n* = 899 (19%)	*n* = 899 (19%)	*n* = 1151 (24%)
Troponin, ng/L (median, range)					
Men	1.9 (1.2–2.0)	3.0 (2.1–4.0)	5.9 (4.1–8.0)	15.0 (8.1–34.0)	484.5 (34.1–50 000)
Women	1.2 (1.2–1.9)	2.0 (2.0–2.9)	3.0 (3.0–6.0)	9.0 (6.1–16.0)	100.0 (16.1–50 000)
Age, years (mean, SD)	49 (13)	58 (14)	66 (14)	71 (14)	71 (15)
Females	378 (42%)	378 (42%)	378 (42%)	378 (42%)	545 (47%)
Diabetes mellitus	68 (9%)	115 (15%)	130 (16%)	162 (21%)	191 (18%)
Hypertension	120 (16%)	224 (29%)	290 (36%)	316 (40%)	444 (41%)
Hyperlipidaemia	119 (16%)	209 (27%)	223 (28%)	238 (30%)	336 (31%)
Ischaemic heart disease	93 (12%)	201 (26%)	304 (38%)	372 (47%)	409 (38%)
Previous myocardial infarction	58 (6%)	120 (13%)	159 (18%)	209 (23%)	244 (21%)
Previous stroke	27 (3%)	48 (5%)	54 (6%)	90 (10%)	107 (9%)
Heart failure at index presentation	0 (0%)	7 (1%)	21 (2%)	83 (9%)	243 (21%)
Current or ex-smoker	315 (57%)	309 (58%)	287 (56%)	252 (59%)	401 (63%)
Admission ACE inhibitor/ARB	88 (16%)	160 (28%)	209 (36%)	221 (42%)	270 (38%)
Admission beta blocker	65 (12%)	108 (19%)	166 (29%)	189 (36%)	236 (33%)
Killip class					
1	784 (99%)	784 (97%)	786 (96%)	699 (88%)	923 (85%)
2	8 (1%)	25 (3%)	30 (4%)	84 (11%)	108 (10%)
3	0	0	0 (0%)	14 (2%)	52 (5%)
4	0	0	0	1 (0%)	3 (0%)
Creatinine, mg/dL (mean, SD)	0.84 (0.14)	0.86 (0.21)	0.91 (0.26)	1.07 (0.63)	1.13 (0.75)
Heart rate, b.p.m. (mean, SD)	79 (18)	78 (20)	78 (20)	83 (24)	86 (29)
Systolic blood pressure, mmHg (mean, SD)	136 (21)	139 (25)	138 (23)	140 (28)	139 (30)

Values are numbers (proportion), except where indicated.

SD, standard deviation; ACE, angiotensin converting enzyme; ARB, angiotensin receptor blocker.

^a^Group 5—all patients with cardiac troponin concentrations >upper reference limit.

### Heart failure hospitalization

Eighty-three patients were hospitalized with heart failure (40/1000 person years) during a total of 2071 person years follow-up. Patients with cardiac troponin concentrations above the URL (Group 5) were more likely to be hospitalized with heart failure than patients with lower troponin concentrations (118/1000 person years vs. 2/1000 in Groups 1 and 2 combined, HR: 47.2, *Table 2*). Similar associations were evident after adjustment for age and sex, and after further adjustment for diabetes mellitus, hypertension, ischaemic heart disease, previous myocardial infarction, systolic blood pressure, and creatinine concentration (*Table 2*).

**Table 2:** Heart failure hospitalization or cardiac death in suspected acute coronary syndrome stratified by troponin concentration

	Group 1	Group 2	Group 3	Group 4	Group 5	All patients
	*n* = 900 (19%)	*n* = 899 (19%)	*n* = 899 (19%)	*n* = 899 (19%)	*n* = 1151 (24%)	*n* = 4748 (100%)
Troponin (ng/L): Men	1.2–2.0	2.1–4.0	4.1–8.0	8.1–34.0	34.1–50 000	1.2–50 000
Troponin (ng/L): Women	1.2–1.9	2.0–2.9	3.0–6.0	6.1–16.0	16.1–50 000	1.2–50 000
Heart failure hospitalization						
Events, *n*	0	2	4	21	56	83
Person years	407	410	390	390	473	2071
Incidence (per 1000 person years)	0	5	10	54	118	40
HR, unadjusted	1		4.1	21.8	47.2	
HR, model 1	1		2.4	9.9	21.4	
HR, model 2	1		3.0	11.7	28.9	
HR, model 1, continuous^a^	2.80 (1.81–4.31)				1.03 (0.96–1.12)	
Heart failure hospitalization or cardiac death						
Events, *n*	0	2	5	29	84	120
Person years	407	410	390	390	473	2071
Incidence (per 1000 person years)	0	5	13	74	178	58
HR, unadjusted	1		5.2	30.2	71.3	
HR, model 1	1		2.7	11.6	27.6	
HR, model 2	1		2.7	14.4	34.1	
HR, model 1, continuous^a^	3.03 (2.05–4.48)				1.03 (0.97–1.10)	

Hazard ratio (95% CI). Model 1 adjusts for age and sex; model 2 additionally adjusts for diabetes mellitus, hypertension, and ischaemic heart disease, previous myocardial infarction, systolic blood pressure at the index presentation, creatinine at the index presentation and an interaction term between ischaemic heart disease and previous myocardial infarction.

HR: hazard ratio.

^a^Analysis of troponin as a continuous variable among patients with troponin levels below the upper reference limit (Groups 1–4) and above the upper reference limit (Group 5).

**Figure 1:**
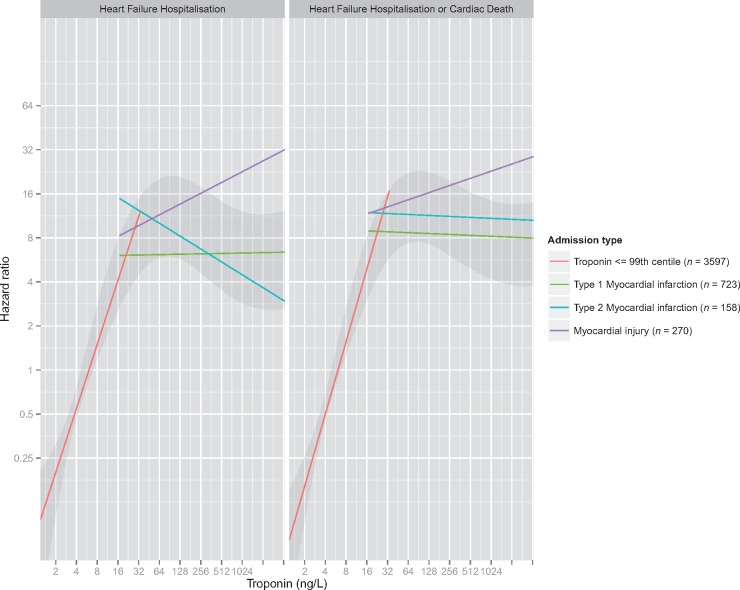
Association between peak cardiac troponin concentration and time to first event for heart failure hospitalization, and heart failure hospitalization or cardiac death. Departures from linearity were explored using penalized spline smoothing functions (grey band). The association was also analysed after stratifying patients into those with a peak troponin concentration less than the upper reference limit (URL) (red line), and those with troponin concentrations above the URL, with a specific index diagnosis (type 1 myocardial infarction, green line; type 2 myocardial infarction, blue; myocardial injury, purple).

In all patients the association between cardiac troponin concentration and heart failure hospitalization was non-linear (*P* for non-linearity <0.001), with a plateau evident at around 30 ng/L as shown via penalized spline smoothing functions (*Figure 1*, Supplementary material online, *Table S2 for model coefficients*).

Among patients with cardiac troponin concentrations below the URL there was a nearly three-fold increase in risk of heart failure hospitalization per doubling of troponin concentration (HR: 2.80, 95% CI 1.81–4.31), whereas among all patients with cardiac troponin concentration above this threshold there was no evidence of an association between increasing cardiac troponin concentrations and heart failure hospitalization (HR: 1.03, 95% CI 0.96–1.12, *Table 2*). On stratifying by the adjudicated diagnosis, patients with type 1 and type 2 myocardial infarction were consistent with negative or weakly positive associations between peak cardiac troponin concentration and hospitalization with heart failure (1.06, 95% CI 0.96–1.17 and 0.81, 95% CI 0.57–1.14, respectively). For patients with myocardial injury there was a positive association (HR: 1.21, 95% CI 1.00–1.47), although this was much weaker than for patients with cardiac troponin concentrations below the URL and the CI included the null (Supplementary material online*, Table S1*). Similar associations were found in analyses using presentation instead of maximal cardiac troponin concentrations (Supplementary material online, *Table S1*).

Similar associations were found in women and men (Supplementary material online, *Figure S1*). The HR in patients with cardiac troponin concentrations below the URL was 3.01 (95% CI 1.50–6.03) for women and 2.68 (95% CI 1.54–4.69) for men. Among patients with troponin concentration above the URL the HR was 1.11 (95% CI 1.01–1.21) for women and 0.88 (95% CI 0.76–1.03) for men.

### Heart failure hospitalization or cardiac death

Similar associations were evident for the combined outcome of hospitalization with heart failure or cardiac death in unadjusted analyses and after adjusting for age and sex, and after further adjustment for diabetes mellitus, hypertension, ischaemic heart disease, previous myocardial infarction, systolic blood pressure, and creatinine concentration (*Table 2* and *Figure 1*).

### Sensitivity analyses

Sensitivity analyses after the exclusion of 354 patients with heart failure [111 patients with troponin <99% centile URL (3.1%), 132 patients with type 1 MI (18.3%), 31 patients with type 2 MI (19.6%), and 80 patients with myocardial injury (29.6%)] identified during the index presentation identified similar associations for cardiac troponin and subsequent heart failure hospitalization, and the composite endpoint of heart failure hospitalization or cardiac death (Supplementary material online, *Tables S2* and *S4*).

### Discrimination

For heart failure hospitalization, cardiac troponin concentration alone achieved similar discrimination to a model incorporating clinical features (age, sex, diabetes mellitus, hypertension and ischaemic heart disease, previous myocardial infarction, systolic blood pressure, and creatinine concentration) (C-statistic 0.80, 95% CI 0.76–0.83 and 0.80, 95% CI 0.75–0.85, respectively, *Table 3*). Moreover, when troponin concentration was added to the risk factors model, the prediction improved to 0.86 (95% CI 0.82–0.89). Similar discrimination was evident for the prediction of heart failure hospitalization or cardiac death (C-statistic 0.87, 95% CI 0.84–0.90). Discrimination was similar in men and women (0.86, 95% CI 0.81–0.91 and 0.87, 95% CI 0.82–0.92, respectively).

**Table 3:** Discrimination of cardiac troponin and a model based on clinical features for heart failure hospitalization or cardiac death

Area under the curve^a^	Troponin^b^	Model^c^	Troponin^b^ + model^c^
Heart failure hospitalization	0.80 (0.76–0.83)	0.80 (0.75–0.85)	0.86 (0.82–0.89)
Men	0.80 (0.75–0.85)	0.81 (0.74–0.88)	0.86 (0.81–0.91)
Women	0.81 (0.77–0.86)	0.80 (0.73–0.87)	0.87 (0.82–0.92)
Heart failure hospitalization or cardiac death	0.81 (0.79–0.84)	0.82 (0.78–0.86)	0.87 (0.84–0.90)
Men	0.82 (0.79–0.86)	0.84 (0.79–0.89)	0.88 (0.84–0.92)
Women	0.82 (0.79–0.86)	0.81 (0.75–0.87)	0.88 (0.84–0.92)

^a^95% CI in brackets.

^b^Including troponin squared.

^c^Model of clinical features age, sex, diabetes mellitus, hypertension, ischaemic heart disease, previous myocardial infarction, systolic blood pressure, and creatinine concentration.

## Discussion

In consecutive patients with suspected acute coronary syndrome high-sensitivity cardiac troponin I concentrations predict an increased risk of subsequent hospitalization with heart failure or cardiac death. Interestingly the relationship between cardiac troponin concentration and heart failure was strongest for patients without myocardial infarction. In these patients, the risk of subsequent hospitalization increased three-fold for every doubling in cardiac troponin concentration and the addition of troponin to a model with clinical features and cardiovascular risk factors markedly improved discrimination.

Heart failure may occur following myocardial infarction in patients with significant myocardial injury and left ventricular systolic impairment. Our observations from a large cohort of consecutive patients with suspected acute coronary syndrome demonstrate that any increase in cardiac troponin concentration <99th centile is associated with an increase in the risk of developing heart failure. Interestingly, in patients with cardiac troponin concentrations >99th centile, further increases in cardiac troponin did not identify those at higher risk. This was true even among patients with type 1 myocardial infarction in whom one might expect more extensive myocardial injury to be associated with a greater risk of heart failure.^19^

The observation that the magnitude of cardiac troponin concentration is not associated with the risk of heart failure in patients with myocardial infarction should perhaps be interpreted with caution, as it has not previously been reported. It is possible that differences in the timing of cardiac troponin measurement could obscure a relationship between troponin concentration and heart failure. In our cohort the majority of patients with troponin concentrations >99th centile had measures at presentation and 12 h (71%) after the onset of symptoms (approximately 6 h following presentation), as recommended by international guidelines for the diagnosis of myocardial infarction.^16^ However, it is now recognized that cardiac troponin concentrations measured during the plateau phase (24–72 h after presentation) are more closely related to the structural sequelae of myocardial infarction as identified on cardiac magnetic resonance imaging, such as scarring and left ventricular impairment, than are troponin concentrations at 6–12 h which are obtained for diagnosis.^20^ An alternative explanation is that other clinical factors are more important than peak troponin concentration in determining whether patients with myocardial infarction develop heart failure, such as the timing and completeness of revascularization, the extent and severity of coronary heart disease, and the presence of comorbid conditions. Competing risks is an unlikely explanation, as similar associations were evident for the composite outcome of heart failure hospitalization or cardiac death as were observed for heart failure hospitalization alone.

Interestingly, in those patients without myocardial infarction where cardiac troponin concentrations were within the normal reference range, troponin concentration was a powerful independent predictor of heart failure hospitalization. Those patients with cardiac troponin concentrations in the lowest two quartiles did not go on to have heart failure or cardiac death. This provides further support to our previous observations that patients with suspected acute coronary syndrome and low cardiac troponin concentrations (<5 ng/L) are at very low risk of cardiovascular events.^13^ It may be that some patients with cardiac troponin concentrations between 5 ng/L and the URL have asymptomatic structural heart disease and are at risk of subsequent decompensation. Echocardiography is recommended in patients with myocardial infarction, but is not routinely performed in those in whom the diagnosis is excluded. Further studies are now needed to determine whether those patients with cardiac troponin concentrations within the normal reference range, but greater than 5 ng/L, would benefit from additional investigations, and treatments to prevent heart failure events.

Indeed, the discrimination of cardiac troponin for predicting subsequent heart failure and cardiac death is excellent.^21^ When added to clinical features and cardiovascular risk factors the area under the receiver operator characteristic score was 0.87, a level indicating that a test is potentially useful for event prediction in individual patients.^22^ Importantly, discrimination was similar in men and women. Previous authors have reported associations between high-sensitivity troponin I/T and heart failure events in the general adult population and in patients with stable coronary artery disease.^23–26^ We now demonstrate that cardiac troponin predicts heart failure admission in unselected patients presenting with suspected acute coronary syndrome, in whom troponin measurement is performed routinely. Our discrimination estimate needs to be validated in an external cohort, but it is unlikely that we have overestimated the C-statistic through over fitting, as only a single continuous variable (troponin) was added to the baseline model.

A strength of this study was that it included all consecutive patients presenting to either secondary or tertiary care hospitals, following referral or self-presentation, in whom acute coronary syndrome was suspected, making this a truly representative sample. However, this approach did mean that the timing of troponin sampling was at the discretion of the attending physician and will have varied by service-related or patient factors. Nonetheless, our findings are generalizable as they reflect usual clinical practice and because they were similar for maximal and presentation cardiac troponin concentrations. A second limitation of this study is that patients were censored at 10 months, and associations between cardiac troponin and heart failure hospitalization or cardiac death at later times could not be determined. However, the first 6-month period is arguably of greatest clinical interest to physicians in preventing subsequent hospitalization. Finally, we do not have N-terminal pro-BNP or BNP concentrations available in this cohort, which would likely further improved the predictive power of our model for heart failure.^23^^,^^27–29^

## Conclusion

High-sensitivity cardiac troponin I is an excellent predictor of heart failure hospitalizations and cardiac death in patients with suspected acute coronary syndrome. Troponin concentrations may, in particular, be used to identify patients without myocardial infarction who are at risk of heart failure. It may be that this group of patients will benefit from N-terminal pro-BNP testing and/or echocardiography. Intervention studies, ideally randomized clinical trials, are needed to determine whether the costs of such a strategy are justified by benefits such as reducing or delaying heart failure admissions.

## Supplementary material

Supplementary material is available at *European Heart Journal – Quality of Care and Clinical Outcomes* online.

## Funding

British Heart Foundation (SP/12/10/29922 and PG/15/51/31596); NHS Scotland Health Informatics Challenge Grant (HICG/1/40) from the Chief Scientists Office; Intermediate Clinical Fellowship by the Wellcome Trust (201492/Z/16/Z to D.M.); Butler Senior Clinical Research Fellowship from the British Heart Foundation (FS/16/14/32023 to N.L.M.); Research Fellowship from Chest Heart and Stroke Scotland (RES/Fell/A163 to A.A.). Abbott Laboratories provided the troponin I assay reagents, calibrators, and controls without charge.

**Conflict of interest**: A.S.V.S. has acted as a consultant for Abbott Laboratories. N.L.M. has acted as a consultant for Abbott Laboratories, Beckman-Coulter, Roche, and Singulex. A.A. and A.R.C. report personal fees from Abbott Diagnostics, outside the submitted work. The other authors declare no competing interests.

## Supplementary Material

Supplementary Data
