# Supplementary material for: High-sensitivity cardiac troponin I and risk of heart failure in patients with suspected acute coronary syndrome: a cohort study
Source: Eur Heart J Qual Care Clin Outcomes. 2017 Jul 19;4(1):36–42. doi: 10.1093/ehjqcco/qcx022 (PMC5805120; doi:10.1093/ehjqcco/qcx022)
Supplement: Supplementary Data [file data_supplement_qcx022.pdf]

SUPPLEMENTARY DATA

High-sensitivity Cardiac Troponin I and Risk of Heart Failure in Patients with Suspected Acute Coronary Syndrome: A Cohort Study

D Stelzle MD MPH[1], ASV Shah MBChB PhD[1], A Anand MBChB[1], FE Strachan PhD[1], AR Chapman MB ChB[1], MA Denvir MBChB PhD[1], NL Mills MBChB PhD[1], DA McAllister MD[2,3]

[1] *BHF Centre for Cardiovascular Science, University of Edinburgh, United Kingdom*
[2] *The Institute of Health and Wellbeing, University of Glasgow, United Kingdom*
[3] *The Usher Institute of Population Health and Informatics, University of Edinburgh, United Kingdom*

Correspondence and requests for reprints:

Dominik Stelzle
BHF Centre for Cardiovascular Science, University of Edinburgh
Edinburgh, EH16 4SB
United Kingdom
Tel: +49 177 1695329
Email: dominik.stelzle@tum.de

Tables and Figures: 5

Support: British Heart Foundation Special Project Grant (SP/12/10/29922), Project Grant (PG/15/51/31596) and Senior Fellowship (FS/16/14/32023)

Table 1 Association of maximal and presentation troponin and the outcomes stratified by admission diagnosis*

	**Troponin ≤99 centile URL**	**Troponin >99th centile URL**			
		**Overall**	**Type 1 MI**	**Type 2 MI**	**Myocardial Injury**
Maximal troponin:					
Heart failure hospitalisation	2.80 (1.81-4.31)	1.03 (0.96-1.12)	1.06 (0.96-1.17)	0.81 (0.57-1.14)	1.21 (1.00-1.47)
Heart failure hospitali-sation or cardiac death	3.03 (2.05-4.47)	1.03 (0.97-1.10)	1.04 (0.96-1.13)	1.00 (0.81-1.24)	1.14 (0.96-1.35)
Presentation troponin:					
Heart failure hospitalisation	2.66 (1.76-4.03)	1.07 (0.97-1.18)	1.06 (0.94-1.20)	0.99 (0.69-1.36)	1.25 (0.98-1.60)
Heart failure hospitali-sation or cardiac death	2.84 (1.97-4.11)	1.05 (0.96-1.14)	1.05 (0.95-1.16)	1.03 (0.81-1.31)	1.12 (0.89-1.41)

* HRs (95%CI), adjusted for age and sex

Figure 1 Association between troponin and time to first event for heart failure hospitalisation and heart failure hospitalisation of cardiac death stratified by gender

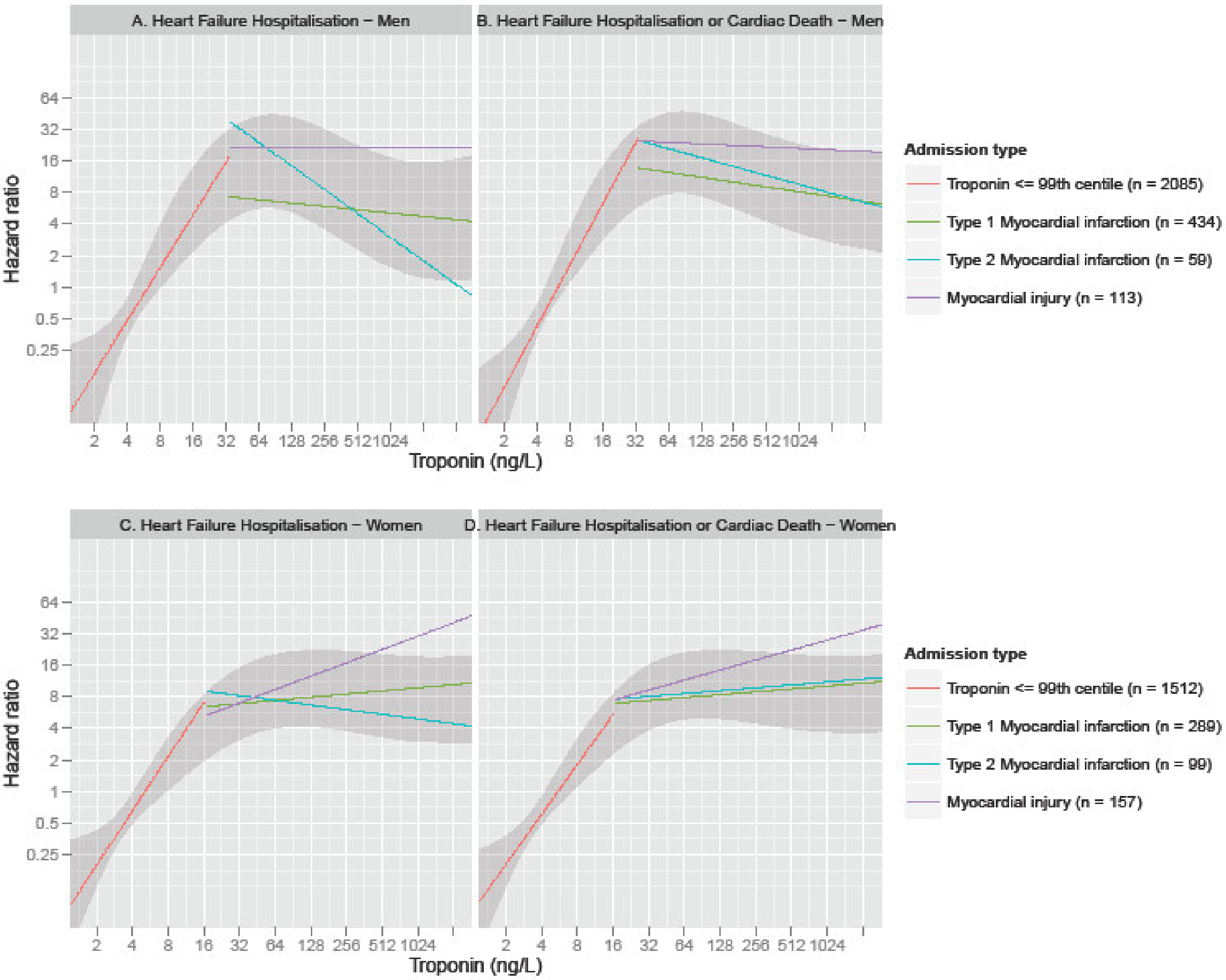

Table 2 Association between troponin and heart failure hospitalisation (or cardiac death) – A comparison of analyses with the complete cohort and sensitivity analyses excluding patients with heart failure at the index presentation

	**Complete cohort**		**Sensitivity analyses**	
	**Troponin**	**Troponin squared**	**Troponin**	**Troponin squared**
Heart failure hospitalisation				
HRs, unadjusted	2.29 (1.82-2.88)	0.96 (0.94-0.97)	2.18 (1.65-2.87)	0.96 (0.95-0.98)
HRs, model 1	1.92 (1.50-2.45)	0.97 (0.95-0.98)	1.87 (1.39-2.52)	0.97 (0.95-0.99)
HRs, model 2	1.84 (1.39-2.43)	0.97 (0.95-0.99)	1.71 (1.23-2.37)	0.98 (0.96-1.00)
Heart failure hospitalisation or cardiac death				
HRs, unadjusted	2.45 (2.01-2.98)	0.95 (0.94-0.97)	2.39 (1.88-3.03)	0.96 (0.94-0.97)
HRs, model 1	1.99 (1.61-2.46)	0.97 (0.96-0.98)	1.99 (1.54-2.57)	0.97 (0.95-0.98)
HRs, model 2	1.92 (1.51-2.45)	0.97 (0.96-0.98)	1.94 (1.45-2.59)	0.97 (0.95-0.99)

Hazard Ratio (95%CI). Model 1 adjusts for age and sex; model 2 additionally adjusts for diabetes mellitus, hypertension, ischaemic heart disease and creatinine concentration at the index admission.

Table 3 Association between troponin and heart failure hospitalisation (or cardiac death) stratified by gender

	Men (n=2691)		Women (n=2057)	
	**Troponin**	**Troponin squared**	**Troponin**	**Troponin squared**
Heart failure hospitalisation (n=36 events in men, n=47 events in women)				
HRs, unadjusted	3.03 (1.97-4.66)	0.94 (0.91-0.96)	2.05 (1.55-2.72)	0.97 (0.95-0.99)
HRs, model 1	2.48 (1.57-3.91)	0.95 (0.92-0.98)	1.75 (1.29-2.37)	0.98 (0.96-1.00)
HRs, model 2	2.41 (1.51-3.85)	0.95 (0.92-0.98)	1.66 (1.20-2.28)	0.98 (0.96-1.00)
Heart failure hospitalisation or cardiac death (n=57 events in men, n=63 events in women)				
HRs, unadjusted	3.48 (2.41-5.03)	0.93 (0.90-0.95)	2.07 (1.62-2.65)	0.97 (0.95-0.98)
HRs, model 1	2.73 (1.84-4.05)	0.94 (0.92-0.97)	1.76 (1.35-2.29)	0.98 (0.96-0.99)
HRs, model 2	2.66 (1.78-3.97)	0.95 (0.92-0.97)	1.69 (1.27-2.25)	0.98 (0.96-1.00)

Hazard Ratio (95%CI). Model 1 adjusts for age; model 2 additionally adjusts for diabetes mellitus, hypertension, ischaemic heart disease and creatinine concentration at the index admission.

Table 4 Association of maximal troponin and the outcomes stratified by admission diagnosis having excluded patients with heart failure*

Sensitivity analysis	Troponin ≤99 centile URL	Troponin >99th centile URL			
		**Overall**	**Type 1 MI**	**Type 2 MI**	**Myocardial Injury**
Maximal troponin:					
Heart failure hospitalisation	2.91 (1.79-4.73)	1.07 (0.97-1.19)	1.08 (0.96-1.22)	0.43 (0.09-2.09)	1.27 (1.00-1.61)
Heart failure hospitalisation or cardiac death	2.97 (1.93-4.58)	1.06 (0.98-1.15)	1.05 (0.95-1.16)	1.14 (0.87-1.49)	1.15 (0.92-1.45)

* Hazard Ratios (95%CI), adjusted for age and sex
